# Intensive management of obesity in people with Prader-Willi syndrome

**DOI:** 10.1007/s12020-022-03064-1

**Published:** 2022-05-07

**Authors:** Brendan J. Nolan, Joseph Proietto, Priya Sumithran

**Affiliations:** 1 Department of Medicine (Austin Health), University of Melbourne, Melbourne, VIC Australia; 2 Department of Endocrinology, Austin Health, Melbourne, VIC Australia; 3 Department of Medicine (St Vincent’s), University of Melbourne, Melbourne, VIC Australia

**Keywords:** Prader-Willi syndrome, Obesity, Weight loss, VLED, Obesity pharmacotherapy

## Abstract

**Purpose:**

Prader-Willi syndrome (PWS) is characterised by childhood-onset hyperphagia and obesity however limited data are available to guide treatment of obesity in this population. We aimed to evaluate the safety, tolerability, and efficacy of intensive medical weight loss interventions (very-low-energy diets [VLED] and/or pharmacotherapy) in individuals with PWS attending a specialist obesity management service.

**Methods:**

A retrospective audit was undertaken of individuals with PWS attending the Austin Health Weight Control Clinic between January 2010-April 2021. Main outcome measures were weight outcomes, duration of use, and adverse effects.

**Results:**

Data were available for 18 patients, of whom 15 were treated with intensive weight loss interventions. Median (interquartile range, IQR) age at baseline was 20 years (19–32) with median body weight 90 kg (75–118) and BMI 37 kg/m^2^ (30–51). Median weight loss during VLED (*n* = 7) was 14 kg (1–20 kg) over 60 weeks. Median weight loss with phentermine-topiramate (*n* = 7) was 17 kg (IQR 9–19 kg) over 56 weeks. Median weight loss with liraglutide 0.6–3 mg (*n* = 7), prescribed with topiramate in 3 individuals, was 9 kg (2–14 kg) over 96 weeks. Naltrexone-bupropion resulted in weight loss in 2 of 4 individuals. Thirteen individuals achieved ≥10% weight loss but only 5 individuals maintained ≥10% weight loss at last follow-up. Five individuals discontinued pharmacotherapy due to adverse effects.

**Conclusions:**

VLED and pharmacotherapy can achieve substantial weight loss in some individuals with PWS though non-adherence results in substantial weight regain. Adverse effects were ascribed to phentermine and topiramate, whereas liraglutide was well-tolerated in this population.

## Introduction

Prader-Willi syndrome (PWS) is a multi-system disorder caused by lack of expression of genes on the paternally inherited chromosome 15q11.2-q13 region. It has an incidence of 1:10,000 to 1:30,000 births. PWS is complicated by hyperphagia with morbid obesity (with resultant risk of type 2 diabetes and obstructive sleep apnoea), intellectual impairment, and short stature. Obesity and its resultant cardiorespiratory comorbidities are major causes of mortality in individuals with PWS [1–3]. Strict dietary supervision and restriction is integral to prevent excessive weight gain. Well-balanced, energy-restricted diets have been shown to improve body weight and body composition in children with PWS [4].

In the general population with obesity, lifestyle interventions comprising diet, physical activity, and behaviour modification, are first-line treatment options. Should weight loss goals not be achieved or maintained, more intensive interventions such as very-low energy diets (VLEDs) plus pharmacotherapy or bariatric surgery should be considered [5]. VLEDs with medical supervision have been shown to be safe and to achieve clinically significant weight loss over 3 years follow-up, with greater and more sustained weight loss associated with addition of obesity pharmacotherapy [6].

Limited data exist on the outcomes of intensive weight loss interventions in individuals with PWS. Case reports and case series have documented successful weight loss following initiation of GLP-1 receptor agonists [7, 8], but data regarding other options are limited. As such, the aims of this retrospective review of real-world clinical data are to examine the efficacy, safety, and tolerability of intensive medical weight loss interventions in individuals with PWS.

## Materials and methods

A retrospective audit of electronic medical records was performed for consultations of patients with PWS attending a multidisciplinary obesity management clinic at Austin Health, Melbourne, Victoria, Australia. The clinic treatment program has previously been described [6]. In short, individuals are prescribed a full VLED program consisting of 3 meal replacement products plus 2 cups of non-starch vegetables (approximately 2300 KJ [550 kcal] per day; 40% protein, 40% carbohydrate and 20% fat), or a partial VLED program consisting of 2 meal replacement products, and one meal consisting of lean protein and 2 cups of non-starch vegetables (approximately 3350 KJ [800 kcal] per day). Where indicated, pharmacotherapy is added for appetite reduction. During the study period, pharmacotherapy included combination phentermine 15–30 mg once daily and topiramate 25–100 mg twice daily, liraglutide 0.6–3 mg once daily, or naltrexone-bupropion 8 mg/90 mg once or twice daily. In our clinic, phentermine-topiramate was frequently prescribed in combination [9] until more recent regulatory approval by the Australian Register of Therapeutic Goods (ARTG) for liraglutide and naltrexone-bupropion during the follow-up period.

With the exception of phentermine and topiramate that were subsidised by the hospital, VLEDs and other pharmacotherapies were largely self-funded. In Australia, the cost of medications indicated for obesity treatment is not subsidised by the government, therefore medications were often used at lower than maximum dosages and off label (topiramate) in some individuals.

Data collected as part of routine clinical practice were extracted from participants’ electronic medical records for consultations between January 2010—April 2021. Patient data (age; sex; weight; body mass index (BMI); type and duration of intensive weight loss interventions; data regarding potential adverse effects) were extracted. Weight outcomes were analysed at the initial consultation, before and after each intervention, at nadir weight, and last follow-up consultation.

Statistical analyses were performed using STATA version 17.0 software (StataCorp. 2021. *Stata Statistical Software: Release 17*. College Station, TX: StataCorp LLC). Data were not normally distributed, so medians and interquartile ranges (IQR) are reported throughout. Wilcoxon-signed rank tests were used to determine differences in body weight and BMI over time. The Spearman rank correlation was used to report correlation *p* < 0.05 was considered statistically significant.

The Austin Health Human Research Ethics Committee approved this activity as an audit (Audit/21/Austin/10), and individual consent was not obtained.

## Results

Eighteen individuals with PWS were treated in the clinic during the study period. Baseline characteristics are shown in Table 1. Thirteen of 18 individuals had obesity at initial consultation, with median baseline weight and BMI 97 kg and 41 kg/m^2^, respectively. Four individuals lived in supported accommodation, with the remainder living in the community. Several individuals had previous treatment with growth hormone during childhood and/or adolescence with one individual continuing growth hormone treatment into adulthood.

**Table 1:** Clinical characteristics

Age at baseline (years)	20 (19–32)
Sex (female, male)	10 (56%):8 (44%)
Follow-up duration (years)	5 (2–8)
Baseline weight (kg)	90 (75–118)
Baseline body mass index (kg/m^2^)	37 (30–51)
Body mass index (kg/m^2^) category (n, %)	
18.5–24.9	1 (6%)
25.0–29.9	4 (22%)
30.0–34.9	3 (17%)
35–39.9	2 (11%)
≥40	8 (44%)
Obstructive sleep apnoea (n, %)	10 (56%)
Type 2 diabetes (n, %)	6 (33%)
Hypertension (n, %)	5 (28%)
Hyperlipidaemia (n, %)	5 (28%)

Values given as median (interquartile range) or number (percentage)

**Fig. 1:**
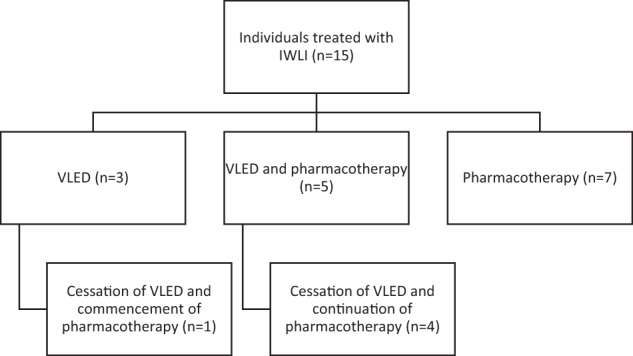
Flowchart of interventions. IWLI intensive weight loss interventions, VLED very low energy diet

During the follow-up period, 15 individuals were treated with an intensive weight loss intervention (Fig. 1). Eight individuals were treated with a VLED, of whom 5 were prescribed concurrent pharmacotherapy. Among those treated with VLED, median peak weight loss was 14 kg (IQR, 1–20 kg, *p* = 0.022 compared to baseline) over median 60 weeks. Five individuals achieved ≥5% weight loss with VLED, 4 of whom were prescribed concurrent pharmacotherapy for appetite reduction. Weight loss was positively correlated with duration of VLED (r = 0.68, *p* = 0.09; or r = 0.84, *p* = 0.04 when not considering one participant who used the VLED for 520 weeks for a weight loss of 15.2 kg).

**Fig. 2:**
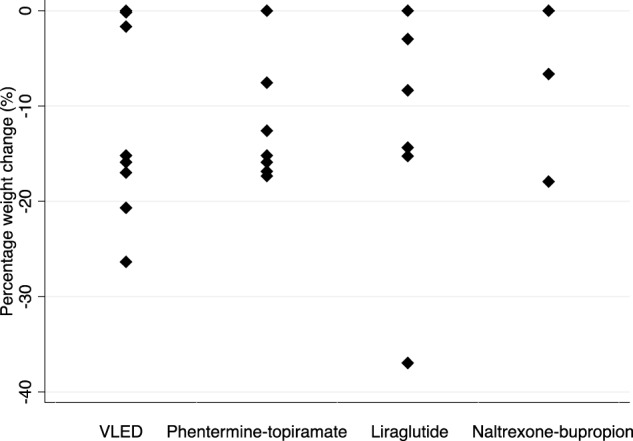
Dot plot representing the maximal percentage weight change associated with VLED and obesity pharmacotherapy. Percentage weight change was calculated at maximal weight loss (nadir weight) with each intervention

In all, 7 individuals were treated with combination phentermine 15–30 mg daily and topiramate 25–100 mg twice daily, 7 individuals were treated with liraglutide 0.6–3.0 mg daily, of whom 3 were prescribed concurrent topiramate, and 4 individuals with naltrexone-bupropion 8/90 mg once or twice daily. Of the 7 individuals prescribed phentermine-topiramate, one continued this at last follow-up, 3 ceased and were subsequently prescribed liraglutide, 2 were subsequently prescribed naltrexone-bupropion, and one ceased pharmacotherapy. Weight loss outcomes are shown in Table 2 and Fig. 2. Adverse effects resulted in discontinuation in five individuals, which were ascribed to phentermine in two patients (insomnia, psychosis), and to topiramate in three (rash, depression, memory impairment). Phentermine was ceased approximately one year after commencement in the individual with psychosis with subsequent resolution. All other adverse effects resulted in discontinuation within 1–18 weeks after commencement. No individual treated with liraglutide ceased due to adverse effects.

**Table 2:** Obesity pharmacotherapy

Medication	Median follow-up (weeks)	Baseline body weight (kg) (median, IQR)	Weight at last follow-up (kg) (median, IQR)	Peak weight loss (median, IQR)	*P* value^$^	Number (%) individuals with ≥ 5% weight loss**	Number (%) individuals with ≥ 10% weight loss
Phentermine 15–30 mg – topiramate 25–100 mg BD^+^	58 weeks	104 kg (82–133)	96 kg (71–115)	17 kg (9–19)	0.001	6/7 (86%)*	5/7 (71%)
Liraglutide 0.6–3 mg^^^	96 weeks	108 kg (104–167)	106 kg (92–133)	9 kg (2–14)	0.12	4/7 (57%)	3/7 (43%)
Naltrexone-buproprion^#^	18 weeks	90 kg (79–120)	90 kg (78–111)	2 kg (0–11)	0.33	1/4 (25%)	1/4 (25%)

^$^*P* value derived from Wilcoxon-signed rank test

^+^Three individuals concurrently treated with VLED

^*^One individual ceased immediately due to insomnia

^^^Prescribed with topiramate in 3 individuals

^#^Added to liraglutide in 2 individuals

**Some individuals were treated with more than 1 ILWI during the follow-up period

**Fig. 3:**
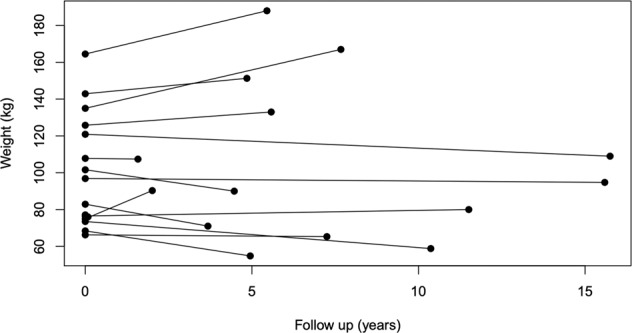
Individual change in body weight from baseline to last follow-up

In the cohort treated with intensive medical weight loss interventions, median peak weight loss was 13 kg (10–15). Fourteen of 15 (93%) of patients achieved ≥5% weight loss at their nadir weight, and 13 (87%) achieved ≥10% weight loss. Treatment non-adherence or discontinuation resulted in weight regain, and median final weight (90 (74–121) vs. 97 (76–123) kg, p = NS) and BMI (38 (31–53) vs. 42 (30–52) kg/m^2^, p = NS) were not significantly different compared to baseline. In all individuals with weight regain, this was attributed to non-adherence with VLED or cessation of pharmacotherapy. Five of 15 individuals maintained ≥10% weight loss at their last follow-up (Fig. 3). Comparing those who achieved weight loss to those with weight gain at their final follow-up, there was a tendency to more successful weight loss in individuals with lower baseline weight, compared to those with a higher baseline weight (*p* = 0.10). Eight individuals had >5 years follow-up. At last follow-up, 1 individual continued treatment with VLED, 1 continued liraglutide and 1 continued naltrexone-bupropion. Three individuals newly commenced treatment with semaglutide, and the remaining 2 individuals were not using a VLED or pharmacotherapy.

No individual underwent bariatric surgery during the follow-up period.

## Discussion

In this retrospective analysis of intensive medical weight loss interventions in adults with PWS attending a specialist obesity treatment service, most (93%) individuals were able to achieve 10% weight loss during treatment but only 5 of 15 maintained ≥10%weight loss at last follow-up visit. In addition to VLED, the majority of patients were prescribed pharmacotherapy for appetite reduction. GLP-1 receptor agonists appear to be well-tolerated in this population.

### Comparison to previous literature

There are currently no data evaluating the use of VLEDs in individuals with PWS. Low carbohydrate diets have been evaluated in a 4-month observational study of 7 children with PWS which found that a modified Atkins diet demonstrated modest 2.9 kg weight loss in one individual, whereas another 3 had weight maintenance [10]. Two individuals were not able to comply with the diet. Similarly in our analysis, several individuals were unable to adhere to the VLED. Potential short-term benefits of a low carbohydrate diet were demonstrated in a 72-hour randomised cross-over trial in 8 children with PWS. Improvements in several metabolic parameters were documented, including decreased postprandial insulin, and a profile of gut hormones favouring control of appetite (increased fasting GLP-1 and GIP, increased postprandial GLP-1 and a decreased ratio of ghrelin:GLP-1), compared to a low-fat diet [11].

GLP-1 receptor agonists are the most studied pharmacological agents in people with PWS. A single-blind randomised cross-over trial of a single dose of exenatide 10 mcg, compared to placebo, demonstrated increased satiety based on an average of 5 repeated visual analogue scale readings from 30 to 240 minutes post-meal in a cohort of 8 individuals with PWS [12]. Case reports and case series [7] with liraglutide [13–15] or exenatide [16, 17] have documented weight loss in individuals with PWS. However, although an improvement in appetite score was reported in a 6-month open-label trial of exenatide 10 mcg twice daily in 10 adolescents and young adults aged 13–25 years, there was no change to BMI over 6 months follow-up (baseline 41.7 kg/m^2^ vs. 6-month 43.1 kg/m^2^) [18].

Here, we report successful weight loss in individuals with PWS treated with combination phentermine and topiramate. To date, one previous case report also reported on the efficacy of phentermine in an adult with PWS [19]. Topiramate has been evaluated in an 8-week randomised-double blind controlled trial of 62 individuals, and no statistically significant weight loss was found (BMI 40.3 to 38.7 kg/m^2^ in topiramate group vs. BMI 41.0 to 40.5 kg/m^2^ in placebo group, *p* = 0.16) [20]. Currently, one case report has found weight maintenance over a six-week period following commencement of naltrexone-bupropion [21].

### Safety considerations

GLP-1 receptor agonists appear well tolerated in individuals with PWS but there are insufficient data to guide the use of other pharmacotherapy options. In keeping with the expected side effect profile of this drug class, nausea was reported in two publications using GLP-1 receptor agonists [17, 18]. Given that PWS is associated with delayed gastric emptying [22, 23], there is a potential that this might be exacerbated by commencement of GLP-1 receptor agonist therapy, though this outcome has not been reported in the literature to date. PWS is associated with impairments in perception of pain and there are reports of gastric rupture and necrosis [24, 25] in people with PWS, therefore a high index of suspicion is required to detect adverse effects after initiation of any new treatment.

In the general population, combination phentermine-topiramate is associated with a high rate of adverse effects including paraesthesia, cognitive changes, headache, dry mouth, and palpitations [9]. However, there are limited data in individuals with PWS. One potential safety consideration is that of psychosis, which has been reported in the general population [26]. This is a theoretical concern in individuals with PWS, in whom emotional dysregulation including temper outburst, depression and psychosis is a cardinal feature [27]. Similarly, individuals with PWS have a higher prevalence of cardiovascular disease compared to the general population, and cardiovascular outcome trial data are currently lacking.

### Limitations

There are several limitations inherent to the retrospective clinical observation including missing data and a lack of untreated individuals to act as a control arm. Similarly, we do not have data on adherence to therapy or the reason for the choice of therapy prescribed. Our audit has small patient numbers; however, this is to be expected given the prevalence of PWS. Despite this, we report long-term follow-up outcomes in which most individuals treated with intensive medical weight loss interventions were able to achieve ≥10% reduction in body weight.

### Future directions

Further long-term controlled trials evaluating VLEDs and obesity pharmacotherapy, including combination pharmacotherapy, in the PWS population are required. The results of a randomised, double-blind placebo-controlled trial of liraglutide in adolescents with PWS are awaited [28]. Newer pharmacological agents including semaglutide 2.4 mg, which has been demonstrated to have clinically significant weight loss in a randomised controlled trial of individuals with overweight or obesity [29], could be evaluated in individuals with PWS.

## Conclusions

In a cohort of patients with PWS, most achieved clinically meaningful weight loss with VLEDs and pharmacotherapy. However, non-adherence resulted in substantial weight regain. Adverse effects were ascribed to phentermine and topiramate and resulted in discontinuation, whereas liraglutide was well-tolerated in this population. Further prospective clinical trials and more effective agents for appetite suppression are required.
